# First identification and characterization of Borrobol‐type tephra in the Greenland ice cores: new deposits and improved age estimates

**DOI:** 10.1002/jqs.3016

**Published:** 2018-02-15

**Authors:** Eliza Cook, Siwan M. Davies, Esther R. Guðmundsdóttir, Peter M. Abbott, Nicholas J. G. Pearce

**Affiliations:** ^1^ Department of Geography Swansea University Swansea UK; ^2^ Centre for Ice and Climate Niels Bohr Institute University of Copenhagen Denmark; ^3^ Nordic Volcanological Center Institute of Earth Sciences University of Iceland Reykjavík Iceland; ^4^ Department of Geography and Earth Sciences Aberystwyth University Aberystwyth UK

**Keywords:** Borrobol, cryptotephra, Greenland ice cores, isochron, Penifiler

## Abstract

Contiguous sampling of ice spanning key intervals of the deglaciation from the Greenland ice cores of NGRIP, GRIP and NEEM has revealed three new silicic cryptotephra deposits that are geochemically similar to the well‐known Borrobol Tephra (BT). The BT is complex and confounded by the younger closely timed and compositionally similar Penifiler Tephra (PT). Two of the deposits found in the ice are in Greenland Interstadial 1e (GI‐1e) and an older deposit is found in Greenland Stadial 2.1 (GS‐2.1). Until now, the BT was confined to GI‐1‐equivalent lacustrine sequences in the British Isles, Sweden and Germany, and our discovery in Greenland ice extends its distribution and geochemical composition. However, the two cryptotephras that fall within GI‐1e ice cannot be separated on the basis of geochemistry and are dated to 14358 ± 177 a b2k and 14252 ± 173 a b2k, just 106 ± 3 years apart. The older deposit is consistent with BT age estimates derived from Scottish sites, while the younger deposit overlaps with both BT and PT age estimates. We suggest that either the BT in Northern European terrestrial sequences represents an amalgamation of tephra from both of the GI‐1e events identified in the ice‐cores or that it relates to just one of the ice‐core events. A firm correlation cannot be established at present due to their strong geochemical similarities. The older tephra horizon, found within all three ice‐cores and dated to 17326 ± 319 a b2k, can be correlated to a known layer within marine sediment cores from the North Iceland Shelf (ca. 17179‐16754 cal a BP). Despite showing similarities to the BT, this deposit can be distinguished on the basis of lower CaO and TiO_2_ and is a valuable new tie‐point that could eventually be used in high‐resolution marine records to compare the climate signals from the ocean and atmosphere.

**Abbreviations**
AMS: accelerated mass spectrometry
CFA: continuous flow analysis
EPMA: electron probe micro‐analysis
GI: Greenland interstadial
LA‐ICP‐MS: laser ablation inductively‐coupled plasma mass spectrometry
MCE: maximum counting error
NA: North Atlantic
REE: rare earth element
SC: similarity coefficient
TA: total alkali
TAU: tephra analysis unit
WD: wavelength dispersive
WDS: wavelength dispersive spectrometry
XRF: X‐ray fluorescence

## Introduction

Tephrochronology has long been established as a tool that exploits ash deposits with unique geochemical fingerprints to precisely correlate a diverse range of palaeoarchives from widely separated localities (e.g. Lowe, 2011). Tephra deposits are preserved in a wide range of depositional environments including marine, ice and terrestrial records and thus have the potential to give rise to valuable time‐synchronous horizons (e.g. Lane *et al*., 2013). Over the last few decades, the scope of this technique has changed considerably through the investigation of cryptotephra deposits that are invisible to the naked eye and can only be detected by employing microscopy techniques (e.g. Davies, 2015). Cryptotephra investigations in Greenland have highlighted the value of polar ice cores as volcanic ash repositories and the potential of bearing isochronous horizons for synchronizing the ice to other palaeoarchives (e.g. Grönvold *et al*., 1995; Mortensen *et al*., 2005; Davies *et al*., 2010, 2008; Abbott and Davies, 2012; Bourne *et al*., 2016, 2015).

**Figure 1:**
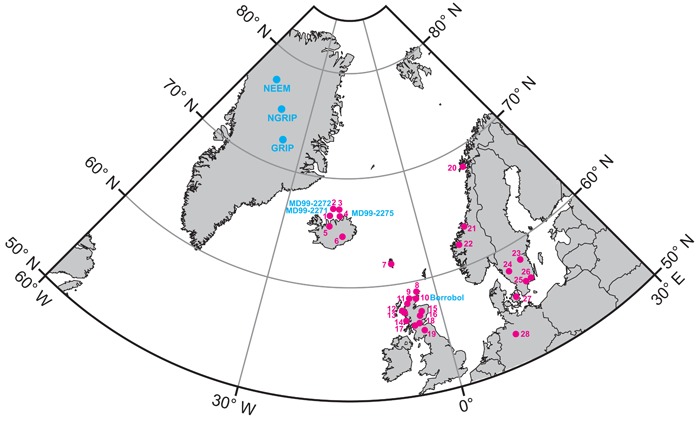
Location of the Greenland ice cores and the distribution of all terrestrial/marine core sites containing Borrobol‐type tephra of GI‐1 and GS‐2 age. Iceland/North Iceland Shelf: (1) MD99‐2271 (Guðmundsdóttir *et al*., 2011). (2) MD99‐2272 (Jarvis, 2013). (3) HM107‐05 (Eiríksson *et al*., 2000). (4) MD99‐2275 (Søndergaard, 2005). (5) Svínavatn (Boygle, 1999). (6) Vatnajökull (Larsen and Eiríksson, 2008). Faroe Islands: (7) Høvdarhagi (Lind and Wastegård, 2011). Orkney: (8) Quoyloo Meadow (Timms *et al*., 2017). Scotland: (9) Lochan An Druim (Ranner *et al*., 2005). (10) Borrobol Bog (Turney *et al*., 1997; Pyne‐O'Donnell, 2007; Pyne‐O'Donnell *et al*., 2008). (11) Tanera Mor (Roberts *et al*., 1998). (12) Druim Loch (Pyne‐O'Donnell, 2007; Pyne‐O'Donnell *et al*., 2008). (13) Loch Ashik (Pyne‐O'Donnell, 2007; Pyne‐O'Donnell *et al*., 2008). (14) Loch an t'Suidhe (Pyne‐ O'Donnell, 2007; Pyne‐O'Donnell *et al*., 2008). (15) Abernethy Forest (Matthews *et al*., 2011). (16) Loch Etteridge (Albert, 2007). (17) Muir Park (Brooks *et al*., 2016). (18) Tynaspirit West (Turney *et al*., 1997; Lowe *et al*., 1999; Pyne‐O'Donnell, 2007; Pyne‐O'Donnell *et al*., 2008). (19) Whitrig Bog (Turney *et al*., 1997; Pyne‐O'Donnell, 2007; Pyne‐O'Donnell *et al*., 2008). Norway: (20) Borge (Pilcher *et al*., 2005). (21) Fosen peninsula (Lind *et al*., 2013). (22) Dimnamyra Bog (Koren *et al*., 2008). (23) Högstorpsmossen (Björck and Wastegård, 1999). (24) Mulakullegöl (Lilja *et al*., 2013). Sweden: (25) Hässeldala port (Davies *et al*., 2003, 2004; Lind *et al*., 2016). (26) Skallahult (Davies *et al*., 2003; Lind *et al*., 2016). Denmark: (27) Østerskov (Larsen, 2014 cited in Lind *et al*., 2016). Germany: (28) Lake Hämelsee (Jones *et al*., 2018).

**Figure 2:**
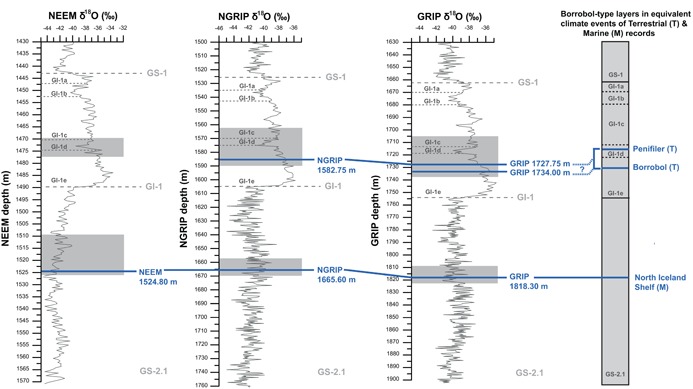
The sampling strategy adopted to search for Borrobol‐type tephra deposits in NEEM, NGRIP and GRIP ice cores, presented against depth and δ^18^O profiles for the interval between GI‐1 and GS‐2.1 (GRIP: Johnsen *et al*., 1997; NGRIP: NGRIP Members, 2004; NEEM: Buizert *et al*., 2014). For each core, the depth intervals sampled are shaded in grey. Horizontal blue bars highlight tephra layers found in each core and proposed tie‐points between cores are shown. The stratigraphic position of three known Borrobol‐type deposits (North Iceland Shelf, BT, PT) found in terrestrial and marine sediments (e.g. Gudmundsdottir *et al*., 2011, 2012; Matthews *et al*., 2011; Brooks *et al*., 2016) is shown for context. References for all sites containing these horizons are given in Fig. 1.

Many Lateglacial tephra deposits identified in European terrestrial records, however, have not yet been identified in the ice. Here we target our searches to identify the Borrobol (BT) and Penifiler (PT) cryptotephras in the Greenland ice cores. Both are distinguishable from other Lateglacial cryptotephras by low FeO and TiO_2_ and high MnO content and are found exclusively in terrestrial deposits in the North Atlantic (NA) region. The BT and PT are close in age and composition and, as a result, present problems for correlation purposes (see Lind *et al*., 2016 for a summary of BT and PT findings in NA records). The BT was first identified in three Scottish palaeolakes, Borrobol Bog, Tynaspirit West and Whitrig Bog by Turney *et al*. (1997) in early Lateglacial Interstadial sediments [probably analogous to Greenland Interstadial 1e (GI‐1e) in Greenland or Bølling in Scandinavia] and was subsequently thought to have been identified at Hässeldala port and Skallahult in Sweden by Davies *et al*. (2003) (see Fig. 1 for site locations). However, with a new pollen stratigraphy and age estimates, Davies *et al*. (2004) showed that the horizon identified in Hässeldala port is associated with Older Dryas sediments (probably analogous to the short‐lived GI‐1d cold event in Greenland). This discovery was inconsistent with the Scottish occurrences that were associated with older Lateglacial interstadial sediments (analogous to the warmer GI‐1e) and prompted Davies *et al*. (2004) to propose that two tephras with identical geochemistry were deposited during GI‐1. Further evidence to support this was presented by Pyne‐O'Donnell (2007) and Pyne‐O'Donnell *et al*. (2008) who revisited the Scottish palaeolakes investigated by Turney *et al*. (1997) and identified two closely spaced horizons with an identical composition to the BT. The deposits were positioned in what were described by the authors as early‐ and mid‐interstadial sediments (Fig. 2) and they recommended that the older deposit should be considered the BT, as defined by Turney *et al*. (1997), while the younger deposit was named the PT. Subsequent work by Matthews *et al*. (2011) outlined new radiocarbon age estimates for the BT and PT horizons based on their preservation within a well‐resolved record from Abernethy Forest, Scotland (Fig. 1). These Bayesian age‐model estimates were updated by Bronk Ramsey *et al*. (2015) and are as follows: BT is 14 098 ± 47 (μ ± 1σ) or 14190–14003 cal a BP (95%; IntCal13), and PT is 13 939 ± 66 (μ ± 1σ) or 14 063–13 808 cal a BP (95%; IntCal13). Both tephra deposits are close in age but a synthesis of age estimates, stratigraphic positions and, more importantly, chironomid‐inferred temperature records led Brooks *et al*. (2016) to conclude that the BT was deposited during the latter stages of GI‐1e. The PT, however, is thought to be associated with a colder interval, probably analogous to GI‐1d. The chironomid‐inferred temperature record from Whitrig Bog provides crucial evidence here as this is the only site, as yet, that fully captures the warming transition at the start of the Lateglacial interstadial (GI‐1) and, as such, constrains the BT to the latter stages of GI‐1e (Brooks and Birks, 2000; Brooks *et al*., 2016; Walker and Lowe, in press). At other Scottish sites a lag in the start of organic sedimentation has been proposed as an explanation for finding the BT at the base of Lateglacial sedimentary profiles and thus misinterpreted as equivalent to early GI‐1e in previous studies (Walker and Lowe, in press).

The occurrence of two separate eruptions with similar ages and identical geochemical compositions means there is a danger of miscorrelation, especially for sites that only preserve a single tephra deposit (e.g. records such as Hässeldala port and Skallahult; Davies *et al*., 2003, 2004). Current thinking suggests a correlation between the Swedish deposits and the PT based on pollen evidence and stratigraphic position, but this cannot be proven given the overlap between BT and PT age estimates (Bronk Ramsey *et al*., 2015). Furthermore, new trace element comparisons of the BT, extracted from a new core from the Borrobol site and (presumably) the PT from these Swedish sites found that the deposits were indistinguishable from each other (Lind *et al*., 2016).

To complicate matters, individual deposits in marine cores HM107‐05 (382–379 cm), MD99‐2275 (3679 cm) and MD99‐2271 (806–808 cm) from the North Iceland shelf have been discovered by Eiríksson *et al*. (2000), Søndergaard (2005) and Gudmundsdóttir *et al*. (2011, 2012), respectively. All cores have been correlated to each other by Gudmundsdóttir *et al*. (2012) using magnetic susceptibility and isotope profiles and the tephra deposits found were originally correlated to the BT based on geochemical similarities (i.e. Haflidason *et al*., 2000; Gudmundsdóttir *et al*., 2011, 2012). However, all tephra deposits are in fact associated with glacial sediments, stratigraphically located below Bølling or GI‐1‐equivalent material. Indeed, the Borrobol‐type tephra (i.e. a similar composition to BT) in HM107‐05 has an age range of 16 490–16 120 cal a BP (±1σ), based on calibrated (CALIB 3.0) accelerated mass spectrometry (AMS) ^14^C dating of benthic foraminifera. Similarly, a Borrobol‐type tephra was found in another core, MD99‐2272 (1697 cm), by Jarvis (2013) and the best available AMS ^14^C age estimate is 17 179–16 754 cal a BP (±1σ), derived from mollusc shell material located 4.5 cm above the tephra layer (calibrated using OxCal 4.1 and the Marine04 curve). Stratigraphic and chronological evidence therefore indicates that the marine layers were deposited in GS‐2.1‐equivalent glacial sediments and this discrepancy between terrestrial and marine‐based radiocarbon age estimates was first noted by Lowe (2001). Both Jarvis (2013) and Eiríksson *et al*. (2004) applied a standard reservoir correction of 400 years to their ^14^C dates, yet Eiríksson *et al*. (2004) noted that the temporal variability in reservoir ages around the North Iceland Shelf exceeds the variability expected from marine model calculations, and may explain offsets between their marine age estimates and between the Icelandic shelf and NGRIP ice‐core age estimates.

Since the first identification of the BT by Turney *et al*. (1997), deposits with a similar composition to the BT have been identified in 28 locations around the NA spanning the early and late Holocene, GI‐1d, GI‐1e and GS‐2.1 (Fig. 1). Here we undertake a comprehensive search of the high‐resolution Greenland ice cores in an attempt to pinpoint the stratigraphic position of the BT, PT and the GS‐2.1 tephra. We also refine the signature of the deposits by major and trace element analysis, particularly to explore whether the latter can aid in discriminating between the different tephras.

## Methodology

### Ice‐core sampling

Three Greenland ice‐cores (NGRIP, GRIP and NEEM) were used to search for the BT, PT and the older Borrobol‐type tephras between GS‐2.1 and GI‐1. The timing of Greenland interstadials (GI) and stadials (GS) and ages presented in this study have been defined by Greenland Ice Core Chronology 2005 (GICC05) (Andersen *et al*., 2006; Rasmussen *et al*., 2006, 2014; Seierstad *et al*., 2014) and GICC05modelext‐NEEM‐1 (Rasmussen *et al*., 2013). The GICC05 multi‐core (NGRIP, DYE‐3, GRIP) timescale was constructed by counting annual layers back from 2000 AD (b2k) using multiple parameters (e.g. δ^18^O, calcium ions) and uncertainty is based on a maximum counting error (MCE) of ambiguous layers, equivalent to 2σ, where cumulative errors increase with depth (Andersen *et al*., 2006; Rasmussen *et al*., 2006). NGRIP and GRIP ice samples were selected to encompass mid‐GI‐1e through to early GI‐1c ice (Fig. 2) to maximize the chances of isolating the BT and PT. The GI‐1 sampling strategy for NEEM was based on coarse‐resolution screening of meltwater samples (1.1 m) derived directly from the continuous flow analysis (CFA) system (Bigler *et al*., 2011) for the entire interstadial and high‐resolution sampling was informed by the age estimates of Matthews *et al*. (2011) and GICC05modelext‐NEEM‐1, which encompassed mid‐ to late GI‐1e and the complete GI‐1d (Fig. 2). To trace the older GS‐2.1‐equivalent Borrobol‐type tephra, coarse‐resolution CFA samples from NEEM were screened and used to inform a higher‐resolution sampling strategy for NEEM, NGRIP and GRIP (Fig. 2). All ice sampling was contiguous to maximize cryptotephra extraction. Ice cores are cut into sections of 55‐cm length in the field, and a 2‐cm^3^ section of ice was cut from the outer edge of each 55‐cm section and further subsampled at a resolution of 15–20 cm. Individual samples were melted and centrifuged in tubes for 5 min at 2500 r.p.m. and at the end of this process any particulate matter, including tephra, remained concentrated at the bottom of the tubes. Supernatant water was discarded, leaving 2–3 mL of sample that was evaporated onto a frosted glass microscope slide and covered in epoxy resin for optical assessment, using high‐magnification light microscopy. Slides containing tephra were selected for electron probe micro‐analysis (EPMA).

### Geochemical analysis

EPMA by wavelength dispersive spectrometry (WDS) is the preferred method for major element characterization of individual tephra grains and requires flat exposed sections through grains for electron bombardment and X‐ray generation (Hunt and Hill, 1993; Hayward, 2012). To obtain these thin sections, epoxy resin was ground down using electrocoated silicon carbide paper and then polished using 6‐, 3‐ and 1‐μm diamond suspension and 0.3‐μm alumina micro polish. EPMA was performed using a Cameca SX100 electron probe microanalyser at the Tephra Analysis Unit, University of Edinburgh. This system has five wavelength dispersive spectrometers and was calibrated daily using internal calibration standards as described by Hayward (2012) and secondary standards were analysed daily and monitored to identify instrumental drift. Major element and secondary standard concentrations are provided in Supporting Information, Table S1.

Trace element analyses were performed on the same glass shards that had been analysed for major elements, using laser ablation inductively‐coupled plasma mass spectrometry (LA‐ICP‐MS) at the Department of Geography and Earth Sciences, Aberystwyth University. Here a Coherent GeoLas ArF 193 nm Excimer LA system was operated with a fluence of 10 J cm^−2^ at a repetition rate of 5 Hz. The analyses were performed using 10‐µm ablation craters, with spectra collected for a 24‐s acquisition on a Thermo Finnegan Element 2 sector field ICP‐MS. The minor ^29^Si isotope was used as the internal standard (using the anhydrous, normalized SiO_2_ from EPMA) with the NIST 612 reference glass used for calibration, taking concentrations from Pearce *et al*. (1997). A fractionation factor was applied to the data to account for analytical bias related to the different matrices of the reference standard and the sample. Data were filtered for inclusion of phenocryst phases to leave only glass analyses. Full details of these methods as well as LA‐ICP‐MS operating conditions are given in Pearce *et al*. (2011) and Pearce (2014) and trace element concentrations are provided in Table S2.

## Results

Tephra deposits were identified within GI‐1e and GS‐2.1 ice. Despite sampling the entire GI‐1d cold event in three ice cores no colourless glass shards consistent with the BT/PT were identified. Other stratigraphically significant cryptotephras were identified within these sampling windows (Fig. 2), some of which were used by Seierstad *et al*. (2014) for the timescale transfer of GICC05 to GRIP; however, this work focuses only on the Borrobol‐type horizons and the full tephrostratigraphy will be reported elsewhere.

### Tephra in GI‐1e ice: stratigraphic position and geochemical composition

**Figure 3:**
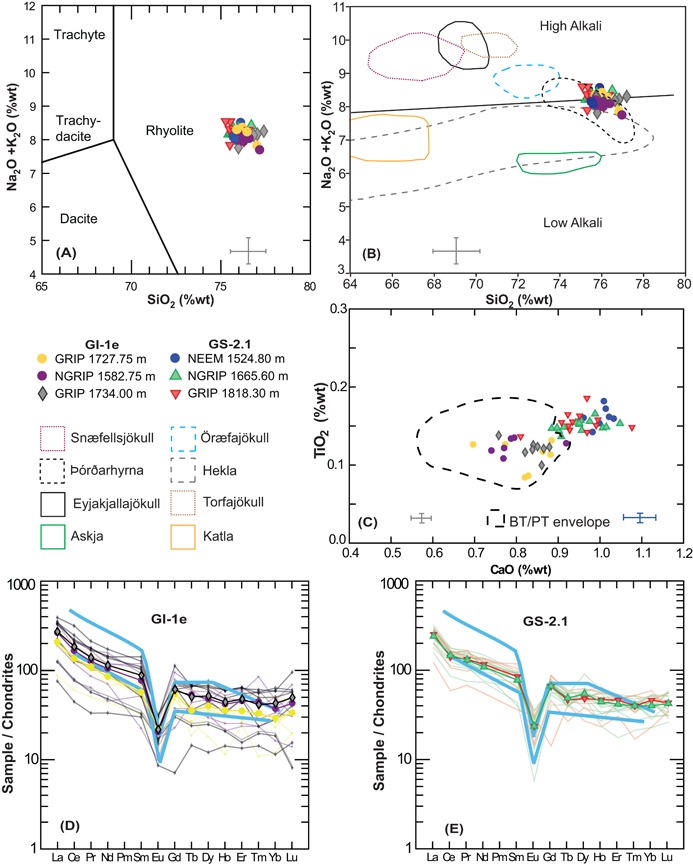
(A) Normalized glass analyses from three GI‐1e and three GS‐2.1 ice core deposits, plotted against rock type, which is assigned on the total alkali *vs*. silica content (TAS) (Le Bas *et al*., 1986). Data are normalized to an anhydrous basis (i.e. 100% total oxides) following the recommendation of Pearce *et al*. (2014). (B) Compositional envelopes for identifying tephra provenance of Icelandic silicic tephra layers using major elements. The diagram is adapted from Meara (2012) with data for Eyjafjallajokull and Snafellsjokull volcanic systems sourced from Larsen *et al*. (1999) and Jonasson (2007). The low and high alkali boundary is based on Jonasson (2007). (C) Comparison of the GI‐1e and GS‐2.1 ice core deposits relative to the compositional envelope for the BT and PT are constructed using data from Turney *et al*. (1997), Pyne‐O'Donnell (2007), Pyne‐O'Donnell *et al*. (2008), Matthews *et al*. (2011) and Lind *et al*. (2016). This comparison highlights the difference in CaO and TiO_2_ in the GS‐2.1 ice‐core tephras. Error bars represent two standard deviations (2σ) of replicate analyses of the Lipari obsidian secondary standard; grey bars correspond to GI‐1e samples and blue to GS‐2.1 samples. (D and E) Chondrite‐normalized REE profiles for individual grains from GI‐1e and GS‐2.1 deposits respectively. The chondrite composition is from McDonough and Sun (1995) and thick blue bars represent end member characterizations of Icelandic rhyolites, reported in Óskarsson *et al*. (1982).

Two individual cryptotephra deposits composed of colourless/pinkish shards with distinctive fluted/cuspate morphology were identified in GI‐1e in GRIP and one deposit in NGRIP (Fig. 2, Table 1). The older of the two GRIP deposits was found at 1734 m depth (14 358 ± 177 a b2k or 14 308 ± 177 a BP) and the younger was found at 1727.75 m depth (14 252 ± 173 a b2k or 14 202 ± 173 a BP). The single GI‐1e deposit in NGRIP was found at a depth of 1582.75 m (14 252 ± 173 a b2k or 14 202 ± 173 a BP). No rhyolitic material was identified in NEEM in the targeted GI‐1e‐d sampling window. Major element analyses (Table 2) show a near identical composition between all these layers which all have a homogeneous population that spans the boundary between low‐ and high‐alkali rhyolites (Fig. 3A,B). The total alkali (TA) content (Na_2_O + K_2_O) ranges between 7.69 and 8.55 wt%, the SiO_2_ values range between 75.90 and 77.40 wt% and the FeO and TiO_2_ contents are between 1.20 and 1.83 wt% and 0.08 and 0.19 wt%, respectively (Table 2; Supporting Table S1; Fig. 3A–C). Statistical analysis of GI‐1e sample pairs found in NGRIP (1582.75 m) and GRIP (1727.75 and 1734 m) supports a common origin from a single volcano, based on high similarity coefficients (SC between 0.979 and 0.981) and low *D* ^2^ values between 0.280 and 1.088, far below the *D* ^2^_critical_ value of 18.48 at the 99% confidence level.

**Table 1:** Summary information for Borrobol‐type tephra deposits from GI‐1e and GS‐2.1 including the ice‐core depth interval (metres) within which each deposit was found and a Greenland Ice Core Chronology 2005 (GICC05) age (using the lower ice depth age). Age uncertainty is based on ‘uncertain annual layers’ and for N uncertain layers the error = N × 0.5 years, and the accumulated error is obtained by summing these layers and is called the maximum counting error (MCE), equivalent to 2σ (Andersen *et al*., 2006; Rasmussen *et al*., 2006). For NEEM, a GICC05 age has been assigned to the GS‐2.1 deposit 1524.80 m as it can be correlated to the NGRIP deposit at 1665.60 m. Geochemical composition, shard concentrations and average shard size are provided. The rock type classification is based on Le Maitre (2002). EPMA conditions were optimized for analysis of small cryptotephra grains (<20 µm diameter) and samples in this study were analysed with either a 5‐ or 3‐μm beam diameter using the operating conditions outlined in Hayward (2012)

Core	Depth (top) (m)	Depth (bottom) (m)	Max. age (a b2k) and MCE	Composition	Grain count	Grain size: long axis (μm)	EPMA beam size (μm)	Event
NGRIP	1582.55	1582.75	14 252 ± 173	Rhyolite	86	30.5	3	GI‐1e
GRIP	1727.55	1727.75	14 252 ± 173	Rhyolite	102	33.1	5	GI‐1e
GRIP	1733.80	1734.00	14 358 ± 177	Rhyolite	93	34.3	3	GI‐1e
NGRIP	1665.40	1665.60	17 326 ± 319	Rhyolite	>2000	47.4	5	GS‐2.1
GRIP	1818.15	1818.30	17 326 ± 319	Rhyolite	431	49.4	5	GS‐2.1
NEEM	1524.60	1524.80	17 326 ± 319	Rhyolite	124	36.8	5	GS‐2.1

**Table 2:** Mean major and trace element values for each tephra deposit with associated standard deviations (1 or 2σ). Major elements were obtained by EPMA of individual grains and mean anhydrous (norm) values are expressed as weight% of sample, together with average values of raw (hydrous) totals. The ice‐core sample depth and the number of analyses (*n*) are given for each deposit. All analyses were performed at the Tephra Analysis Unit (TAU), University of Edinburgh, using a Cameca SX100 electron microprobe. Trace element data are expressed in parts per million. All samples were analysed by LA‐ICP‐MS at the University of Aberystwyth with a 10‐ μm beam spot size using a Coherent Geolas ArF 193‐nm Eximer laser ablation unit coupled to a Thermo Finnigan Element 2 high‐resolution sector mass spectrometer. The USGS reference glass BCR2‐G was analysed as an unknown under the same operating conditions at the same time. Analytical precision is typically between ±5 and 10% and accuracy is typically around ±5%, when compared with the published concentrations for BCR2‐G

	NGRIP 1582.75 m		GRIP 1727.75 m		GRIP 1734 m		NGRIP 1665.60 m		GRIP 1818.30 m		NEEM 1524.80 m		MD99‐2271 808 cm	
Major elements	Mean (*n* = 6)	2σ	Mean (*n* = 7)	2σ	Mean (*n* = 8)	2σ	Mean (*n* = 25)	2σ	Mean (*n* = 12)	2σ	Mean (*n* = 8)	2σ	Mean (*n* = 8)	2σ
SiO_2_	76.37	0.94	76.48	0.80	76.42	1.18	75.94	0.55	75.83	0.77	75.91	0.29	75.85	2.17
TiO_2_	0.12	0.02	0.11	0.04	0.12	0.02	0.15	0.01	0.15	0.03	0.16	0.02	0.15	0.03
Al_2_O_3_	12.89	0.75	12.61	0.50	12.64	1.14	12.91	0.43	12.89	0.33	12.96	0.42	13.12	1.47
FeO	1.52	0.26	1.46	0.76	1.47	0.31	1.59	0.21	1.60	0.34	1.58	0.18	1.52	0.34
MnO	0.04	0.02	0.04	0.02	0.04	0.03	0.04	0.01	0.04	0.01	0.04	0.01	0.04	0.01
MgO	0.07	0.05	0.09	0.07	0.07	0.04	0.11	0.03	0.11	0.05	0.12	0.05	0.11	0.06
CaO	0.80	0.13	0.82	0.13	0.84	0.08	0.95	0.10	0.95	0.13	1.00	0.06	1.04	0.64
Na_2_O	4.08	0.23	4.16	0.32	4.16	0.39	4.27	0.20	4.28	0.40	4.22	0.30	4.36	0.65
K_2_O	3.90	0.19	3.95	0.22	3.99	0.15	3.91	0.17	3.92	0.22	4.01	0.15	3.79	0.47
P_2_O_5_	0.01	0.01	0.01	0.02	0.01	0.01	0.02	0.01	0.02	0.01	0.02	0.01	0.02	0.01
Cl	0.19	0.17	0.26	0.21	0.24	0.52	0.17	0.15	0.22	0.27	–	–	–	–
**Total**	**98.53**	**2.75**	**98.57**	**2.88**	**98.40**	**1.69**	**98.66**	**2.16**	**98.20**	**2.32**	**97.84**	**2.93**	**96.02**	**1.66**

Trace elements	Mean (*n* = 6)	1σ	Mean (*n* = 5)	1σ	Mean (*n* = 7)	1σ	Mean (*n* = 13)	1σ	Mean (*n* = 7)	1σ	Mean (*n* = 0)	1σ	Mean (*n* = 0)	1σ
Rb	80.63	16.07	68.39	14.40	74.17	21.91	124.25	25.94	109.89	24.04	–	–	–	–
Sr	59.25	5.94	51.96	4.15	59.52	8.32	66.41	7.70	76.75	17.46	–	–	–	–
Y	68.00	25.05	56.85	21.63	76.53	47.84	71.25	18.78	66.96	17.19	–	–	–	–
Zr	293.91	28.91	267.02	25.09	300.65	56.88	267.42	30.87	289.24	44.65	–	–	–	–
Nb	32.54	12.68	25.75	10.54	34.91	19.55	34.48	3.85	30.15	8.88	–	–	–	–
Cs	0.85	0.45	0.87	0.35	0.90	0.41	1.59	1.38	1.34	0.48	–	–	–	–
Ba	675.39	116.72	602.12	102.06	688.67	209.58	479.85	61.21	507.73	90.86	–	–	–	–
La	60.83	21.78	47.79	17.72	60.00	31.14	57.65	11.53	60.45	15.64	–	–	–	–
Ce	98.57	35.30	80.41	29.53	105.55	64.50	90.57	16.22	87.16	25.52	–	–	–	–
Pr	11.68	4.19	10.12	3.64	12.19	6.86	12.53	2.48	12.76	3.49	–	–	–	–
Nd	47.62	18.69	38.72	13.37	49.51	26.47	51.38	10.57	55.01	13.69	–	–	–	–
Sm	11.50	5.76	8.49	2.34	11.67	6.57	12.02	2.94	13.16	4.32	–	–	–	–
Eu	1.16	0.45	1.29	0.56	1.16	0.53	1.39	0.49	1.39	0.44	–	–	–	–
Gd	11.79	6.09	10.95	3.77	11.90	7.46	13.81	2.33	13.71	5.55	–	–	–	–
Tb	1.94	0.53	1.29	0.60	1.73	0.90	1.85	0.31	1.77	0.40	–	–	–	–
Dy	11.85	3.70	9.83	4.81	12.37	6.55	14.05	3.66	12.40	3.24	–	–	–	–
Ho	2.31	0.95	1.97	1.03	2.38	1.21	2.54	0.67	2.67	0.92	–	–	–	–
Er	7.33	2.59	5.67	2.12	7.45	4.05	6.98	1.71	7.79	1.17	–	–	–	–
Tm	1.11	0.63	0.82	0.36	0.96	0.52	1.03	0.21	1.03	0.32	–	–	–	–
Yb	6.16	2.14	4.86	2.48	6.49	3.60	6.89	1.70	7.95	1.93	–	–	–	–
Lu	1.06	0.50	0.83	0.31	1.17	0.82	1.09	0.28	1.11	0.28	–	–	–	–
Hf	10.16	1.46	9.47	1.75	10.05	3.08	9.52	1.64	9.87	1.87	–	–	–	–
Ta	2.81	1.32	2.44	0.94	2.90	1.67	2.94	0.74	2.91	1.30	–	–	–	–
Th	2.73	0.98	2.56	1.07	2.79	1.50	13.83	2.82	16.41	7.18	–	–	–	–
U	57.74	21.18	57.92	2.35	25.11	3.30	3.40	0.68	3.10	1.00	–	–	–	–
Pb	12.09	4.19	10.23	3.36	13.26	7.57	27.14	14.86	27.35	18.91	–	–	–	–

The Icelandic system producing Borrobol‐type material remains unknown (Lind *et al*., 2016) and our major element comparisons indicate that this tephra has no consistent overlap with rhyolitic products of Icelandic origin (Fig. 3B). The exception, however, is the eastern rift zone central volcano ÞórÐarhyrna that has a similar composition (although the reference data are based on just three analyses of whole rock samples). Little is known about this volcano, located beneath the Vatnajökull ice cap, but three nunataks were analysed by X‐ray fluorescence (XRF) by Jónasson (2007) and the limited data are used for comparison in Fig. 3B.

Both GRIP GI‐1e deposits geochemically match the NGRIP deposit, and their chronological positions were assessed by Seierstad *et al*. (2014) as part of a wider study to identify tie‐points between cores for transfer of the GICC05 chronology to the GRIP record. The two youngest deposits, NGRIP 1582.75 m and GRIP 1727.75 m, were matched and thus share the common age of 14 252 ± 173 a b2k and also occupy a stratigraphic position approximately 200 years before the start of GI‐1d. GRIP 1734 m is 106 ± 3 years older (14 358 ± 177 a b2k) and is found in the middle of the GI‐1e warm event, immediately before a gradual downturn trend in surface air temperature according to the δ^18^O record (Fig. 2) (NGRIP Members, 2004).

**Figure 4:**
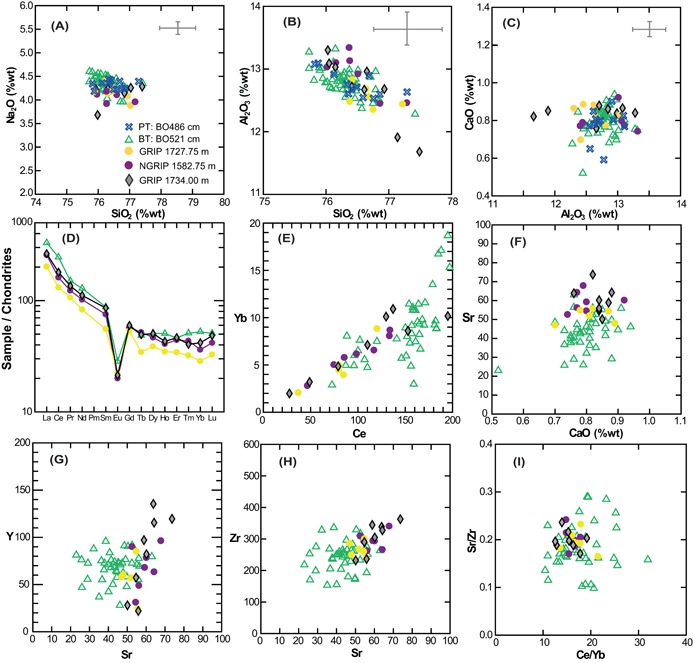
(A–C) Comparison of GRIP 1734 m, GRIP 1727.75 m and NGRIP 1582.75 m major element data against BT (BO521) and PT (BO486) data from Lind *et al*. (2016). Error bars in all diagrams represent one standard deviation (1σ) of replicate analyses of the Lipari obsidian secondary standard. (D) Single‐shard and average chondrite‐normalized REE profiles from GRIP 1734 m, GRIP 1727.75 m and NGRIP 1582.75 m ice cores, against BT data from Lind *et al*. (2016). (E–H) Element‐to‐element biplots for trace elements based on absolute concentrations (p.p.m.) from single grains and (I) ratios.

Single‐shards were analysed by LA‐ICP‐MS (Table 2) and when average rare earth element (REE) profiles are displayed together with the individual grain analyses, there is general similarity between all layers from GI‐1e (Fig. 3D). The REE profiles appear typical of Icelandic rhyolitic products based on a comparison with end‐member characterizations of Icelandic rhyolites from Óskarsson *et al*. (1982) (Fig. 3D). This includes high absolute concentrations of Sr, Zr and Ba, light REE (LREE) enrichment (La to Nd >100 times the chondritic value) with a profile that slopes steeply down to the pronounced negative anomaly of Eu, indicating feldspar fractionation. The steep profile of these incompatible LREEs gives way to a flat profile that characterizes the abundance of middle REEs (MREEs) and heavy REEs (HREEs) between Gd and Lu. The range of concentrations and element ratios, *e.g*. Ce/Yb (Fig. 4) are the same for sample pairs GRIP 1727.75 m and NGRIP 1582.75 m, and also GRIP 1734 m, although not identical in terms of REE, with the NGRIP sample looking to be more evolved than GRIP 1727.75 m, based on higher REE abundance. It must be emphasized that only a small number of analyses were possible on the Greenland ice‐core samples (Table 2) and these may only represent part of the eruption's compositional range, with the possibility that further analyses could extend the fields of data. When coupled with the analytical noise for analyses performed at 10 μm, close to the limit of the LA‐ICP‐MS method, it should be noted that the data will be influenced by larger uncertainties that do not typically hamper analyses of larger particles. Statistical analysis of 15 trace elements from deposits NGRIP 1582.75 m and GRIP 1727.75 m (that form the younger GI‐1e horizon) produces a *D* ^2^_critical_ value of 3.506, which is below the critical value of 30.58 at the 99% confidence level and demonstrates that the geochemical composition is not significantly different.

### Geochemical and chronological comparison to other North Atlantic Borrobol‐type deposits in GI‐1e

The two ice‐core tephra horizons fall within the BT/PT compositional envelope (Fig. 3C) and the best geochemical matches are with the new data‐sets (BT: BO521 and PT: BO486) from the original Borrobol site published in Lind *et al*. (2016) (Fig. 4A–C). Older data have a consistently lower Na_2_O content, typically ∼0.65 wt% less than the ice‐core results. The offset is probably due to sodium loss in older analyses and the similarities between our data and the Lind *et al*. (2016) analyses could be because they were both analysed with improved conditions and EPMA modification, described by Hayward (2012). There is consistent major element overlap between the ice‐core data and BO521 and BO486 and all exhibit the trend of evolution by fractionation of feldspar (Fig. 4B), which is typical for Icelandic rhyolites (SiO_2_ increases as CaO and Al_2_O_3_ decrease). Lind *et al*. (2016) have assumed BT and PT ages from Bronk Ramsey *et al*. (2015), so with respect to the ice‐core age estimates, the older ice deposit GRIP 1734 m (14 308 ± 177 a BP) is consistent only with the BT (14 190–14 003 cal a BP). Statistical investigation of the dataset shows compositional similarity between these two layers, with SC and *D* ^2^ values of 0.974 and 1.614, respectively (Table 3). The younger Greenland deposit (NGRIP 1582.75 m/GRIP 1727.75 m; 14 202 ± 173 a BP) overlaps on age with both the BT and the PT (14 063–13 939 cal a BP) with SC and *D* ^2^ values of 0.966 and 3.036 and 0.974 and 4.527, respectively (Table 3).

**Table 3:** Graphical comparisons between major and trace element datasets were supported by two statistical tests; the similarity coefficient (SC) of Borchardt *et al*. (1972) and statistical distance (*D* ^2^) method of Perkins *et al*. (1998, 1995). This table presents SC and *D* ^2^ values for major elements (normalized to 100%), and *D* ^2^ values for trace elements (T). Five major elements (with >1 wt%) were used for SC calculations, based on the method from Hunt *et al*. (1995), where values >0.95 suggest products are from the same volcanic source. *D* ^2^ is from Perkins *et al*. (1998, 1995) and seven major elements were used in the comparisons (with >0.01 wt%). The value for testing the statistical distance values at the 99% confidence interval is 18.48 (seven degrees of freedom). For calculating *D* ^2^, 15 trace elements were used, following recommendations by Pearce *et al*. (2008). The value for testing *D* ^2^ at the 99% confidence interval is 30.58 (15 degrees of freedom)

Major and trace element similarity: GI‐1e Borrobol‐type		
Deposit	BT: BO521	PT: BO486
GRIP 1727.75 and NGRIP 1582.75 m	SC 0.966	SC 0.974
	*D* ^2^ 3.036	*D* ^2^ 4.527
	*D* ^2^ 10.078 (T)	
GRIP 1734.00 m	SC 0.974	SC 0.987
	*D* ^2^ 1.614	*D* ^2^ 1.131
	*D* ^2^ 5.137 (T)	
Major element similarity: GS‐2.1 Borrobol‐type		
Deposit	MD99‐2271	MD99‐2272
NGRIP 1665.60 m	SC 0.990	SC 0.951
	*D* ^2^ 0.836	*D* ^2^ 4.225
GRIP 1818.30 m	SC 0.990	SC 0.952
	*D* ^2^ 0.725	*D* ^2^ 3.865
NEEM 1524.80 m	SC 0.986	SC 0.957
	*D* ^2^ 2.022	*D* ^2^ 3.479

All ice‐core samples have lower REEs when compared to BO521 (Fig. 4D) although the range of REE patterns (Fig. 4D), trace element concentrations (Fig. 4E–H) and ratios are similar (e.g Ce/Yb in Fig. 4I), which strongly suggests a cogenetic relationship between the layers. Trace elements could not be derived from BO486 (Lind *et al*., 2016). BO521 is slightly more compositionally evolved than the ice‐core samples which have higher CaO and Sr (e.g. Fig. 4F) and a regression line through these analyses (*r* ∼ 0.35) shows Sr decreasing with CaO, consistent with a possible genetic link between them by feldspar extraction. Additionally, almost all the other incompatible elements (e.g. U, Nb, Ta, the REE, and Rb and Ba which behave incompatibly or neutrally in rhyolites) increase from the ice‐core layers to BO521. This suggests the relationship between these samples is related to an eruption from a compositionally zoned or stratified magma chamber, with the more evolved upper part of the magma body producing the BO521 deposit, and later erupted (less evolved) magma from deeper in the magma body travelling to Greenland to be deposited as GRIP 1727.75 m/NGRIP 1582.75 m or GRIP 1734 m. Deposits from the younger ice‐core layer GRIP 1727.75 m/NGRIP 1582.75 m overlap with BO521 in terms of their Sr and Y concentrations (albeit at the less evolved end of the BO521 composition) (Fig. 4G). In contrast, Y is visibly higher in some of the shards from the older ice‐core deposit GRIP 1734 m and this difference may tentatively indicate that GRIP 1734 m and BO521 are not the same, and were produced by different eruptions (Fig. 4G). However, these observations are based on a small number of analyses, and additional analyses are required to explore this further.

### Tephra in GS‐2.1 ice: stratigraphic position and geochemical composition

**Figure 5:**
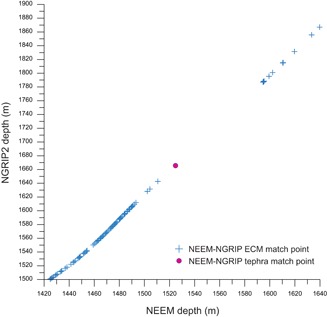
Individual ECM match points between NEEM and NGRIP over the interval of GS‐1 to GS‐3, highlighting a wide gap in GS‐2.1 chemostratigraphic matching (Rasmussen *et al*., 2013). The new GS‐2.1 Borrobol‐type match‐point NEEM 1524.80 m/NGRIP 1665.60 m is shown within this gap.

Three deposits were identified within GS‐2.1 ice in the following samples: NEEM 1524.80 m, NGRIP 1665.60 m and GRIP 1818.30 m (Table 1) and contain high concentrations of colourless glass shards. The ages of the deposits are consistent between cores and the NGRIP 1665.60 m/GRIP 1818.30 m match‐point is included within an NGRIP/GRIP timescale synchronization performed by Seierstad *et al*. (2014). While the GICC05 age for this GS‐2.1 deposit is 17 326 ± 319 a b2k (17 276 ± 319 a BP), the age according to the first NEEM timescale is 17 386 ± 173 a b2k (GICC05modelext‐NEEM‐1). However, this new deposit sits along a trend line (Fig. 5) when plotted together with the depths of NEEM/NGRIP ECM match‐points (from Rasmussen *et al*., 2013), supporting the correlation, and providing a new match‐point to amend GICC05modelext‐NEEM‐1 in a future version of this timescale.

All deposits have an identical rhyolite major element composition (Table 2, Fig. 3A,B) and are almost identical in composition to the GI‐1e ice‐core deposits. It is apparent, however, that there are consistent differences in the CaO and TiO_2_ values that discriminate between the GS‐2.1 and GI‐1e deposits (Fig. 3C). Statistical analyses of major elements support a correlation between the NGRIP, GRIP and NEEM deposits with SC values ranging between 0.988 and 0.995 and *D* ^2^ values ranging between 0.240 and 1.100, strongly suggesting a compositional/genetic link between the deposits.

The average REE profiles of the GS‐2.1 deposits fall within boundaries of typical Icelandic rhyolitic products and are very similar with a particularly good agreement between the incompatible LREE and MREE profiles, including a pronounced negative Eu anomaly (Fig. 3E). There is more variability between individual analyses of HREEs because of the low concentrations of these elements, which are close to detection limits at the analysis crater diameter used here, but this is smoothed out in the averages. Statistical comparison of the GS‐2.1 trace element data of NGRIP 1665.60 m and GRIP 1818.30 m produces a low *D* ^2^ value of 1.401, and this further supports the correlation. It was not possible to obtain reliable trace element data from the NEEM sample because of the small sample size and low signal.

### Geochemical and chronological comparison to other North Atlantic Borrobol‐type deposits in GS‐2.1

**Figure 6:**
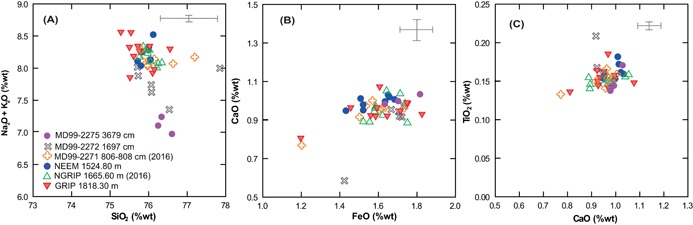
(A–C) Compositional variation diagrams for GS‐2.1 rhyolite deposits of NEEM 1524.80 m, GRIP 1818.30 m and NGRIP 1665.60 m against North Iceland Shelf data from cores MD99‐2271 (Gudmundsdottir *et al*., 2011), MD99‐2271, MD99‐2272 and MD99‐2275 (this study). Error bars are 2σ of replicate analyses of the Lipari obsidian secondary standard.

The GS‐2.1 ice‐core horizon has potential counterparts in the marine realm, with four similar Borrobol‐type deposits found in glacial (GS‐2.1 equivalent) sediments. The best (reservoir‐corrected) age estimate for this marine isochron is 17 179–16 754 cal a BP (Jarvis, 2013) and is comparable to the ice‐core age of 17 276 ± 319 a BP. Furthermore, major element comparisons between the ice‐core deposits and MD99‐2271 (newly acquired data for this study, Table 2), MD99‐2272 and MD99‐2275 (Table S1) shows good agreement between the datasets (Fig. 6). This relationship is supported by statistical analyses, particularly with MD99‐2271, which has an SC of 0.99 and a *D* ^2^ of 2.07 and can be interpreted as a volcanic event match as well as a provenance match. SC and *D* ^2^ values of 0.95 and 4.32, respectively, also support a common volcanic source between the ice‐core deposits and MD99‐2272 (Table 3), although there is an observed Na_2_O offset that probably reflects sodium loss during EPMA (Fig. 6A). A similar offset is observed in data from MD99‐2275 grains, which otherwise compares well to the ice‐core data (Fig. 6B,C). However, with just three analyses from this deposit and low oxide totals, statistical comparison could not be performed with confidence.

## Discussion

Three cryptotephra deposits with a Borrobol‐type composition have been identified for the first time in Greenland ice spanning GS‐2.1 (one horizon) and GI‐1e (two horizons), but Borrobol‐type deposits were absent from GI‐1d ice. For the two compositionally identical events, *ca*. 106 years apart in GI‐1e ice, the correlation issues that plague the BT remain. Without any diagnostic geochemical features, pinpointing a correlation to either the BT or the PT in terrestrial sequences is limited. Our new trace element data show some tentative and subtle differences but require further exploration to robustly assess their use for discrimination purposes. Furthermore, while the older ice‐core deposit (GRIP 1734 m; 14 308 ± 177 a BP) is consistent only with the calibrated age range of the BT, the younger deposit (GRIP 1727.75 m/NGRIP 1582.75 m; 14 202 ± 173 a BP) overlaps with the age ranges of both the BT and the PT layers. Although a firm correlation is precluded, we discuss various possibilities below that will require testing in future work.

**Figure 7:**
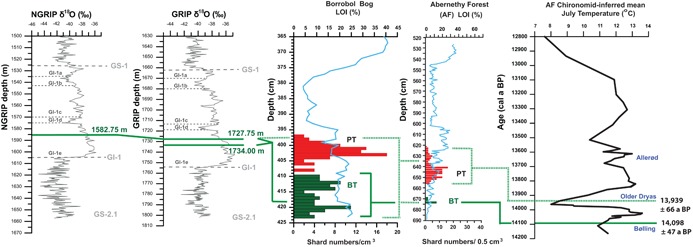
Stratigraphic position of GI‐1e tephra deposits, GRIP 1734 m, GRIP 1727.75 m and NGRIP 1582.75 m against δ^18^O records and potential BT and PT tie‐points in Borrobol and Abernethy Forest cores, Scotland. Loss on ignition (LOI) and shard concentration data are from Pyne‐O'Donnell *et al*. (2008) and Matthews *et al*. (2011). The chironomid‐inferred mean July temperature record from Abernethy Forest is shown to highlight the relative positions of the BT and PT (Brooks *et al*., 2012) with ages from Bronk Ramsey *et al*. (2015). The solid green line represents a scenario where the PT is absent in the Greenland ice and the two GI‐1e ice deposits have been fused into a single diffuse layer in the terrestrial record, potentially shown in the wide Borrobol Bog shard profile. The dashed green line represents another possible scenario where the two ice‐core deposits correlate to the PT and BT and would suggest time‐transgressive changes between Scotland and Greenland.

One possibility is that the GI‐1e deposits in the ice represent two closely spaced eruptions that have become ‘fused’ into one BT deposit in some terrestrial records. Indeed, Pyne‐O'Donnell *et al*. (2008) previously alluded to this after observing diffuse BT shard distributions over 10 cm within the cores from Borrobol Bog (green bars, Fig. 7), Loch an t'Suidhe and Tanera Mor (Roberts *et al*., 1998). A dispersed shard concentration profile is not observed at all sites, however, and a distinct single peak spanning just a few centimetres is observed at Abernethy Forest (Fig. 7) (Matthews *et al*., 2011). The best age estimate of 14 190–14 003 cal a BP for the BT is derived from the latter site by Bronk Ramsey *et al*. (2015) and this age range agrees well with the youngest deposit found in the Greenland ice, but also shows some overlap with the upper age range of the older deposit. This is consistent with our tentative observation that fractional crystallization of feldspar and zircon links BO521 (i.e. BT) more favourably with GRIP 1727.75 m/NGRIP 1582.75 m than GRIP 1734 m, based on higher concentrations of elements such as Y and Al in the latter.

It is therefore possible that some terrestrial sites preserve the GI‐1e tephra couplet as a diffuse unit (e.g. Borrobol Bog), whereas other sites (e.g. Abernethy Forest) may only preserve one of these ice‐core deposits (Fig. 7). The ability to temporally resolve closely spaced volcanic events is a strength afforded to high‐resolution ice cores and, in this context, creates a need to reinvestigate terrestrial samples in ultra‐fine resolution, to explore the finer anatomy of the BT in terrestrial records. This, however, may not be possible due to the relatively lower resolution of terrestrial records. Nevertheless, based on our current data sets, we can suggest that any BT deposit found in Lateglacial terrestrial records should be synchronized to both ice‐core deposits spanning a 106‐year interval. This proposed correlation is consistent with the Scottish chironomid‐inferred temperature record from Whitrig Bog that indicates the BT deposition occurred during an interval that equates with the late GI‐1e in Greenland (Brooks and Birks, 2000; Walker and Lowe, in press) (Fig. 7). However, what we cannot rule out is that climatic changes between Greenland and Scotland during GI‐1 were time‐transgressive, meaning that we cannot rely on climatostratigraphic constraints to support our tephra correlations. We assume that the PT is absent in Greenland as we did not identify any tephras of similar composition within GI‐1d ice. An alternative scenario, however, is that ash from both the BT and the PT were instead deposited in Greenland during GI‐1e, as the older GRIP 1734 m and younger GRIP 1727.75 m/NGRIP 1582.75 m deposit, respectively. We believe that this scenario is unlikely given the implied prolonged delay in climatic response between Greenland and Scotland (Fig. 7). However, we stress the ultimate goal here of employing the BT and PT as independent marker horizons without having to rely on stratigraphic positions to aid and support a correlation. This is a significant challenge given the complexity associated with the BT and PT but some promising signs are presented in relation to the trace element signatures. We urgently need to strive for better geochemical fingerprints to discriminate between the BT and PT so that potential correlations to the ice can be tested.

The tephra identified in GS‐2.1 is simpler in terms of its wider application as an ice‐marine tie‐point. This is the oldest known deposit with a Borrobol‐type composition, but we demonstrate that it can be separated from the BT and PT on the basis of CaO *vs*. TiO_2_ content (Fig. 3C). This compositional difference will be valuable in poorly resolved marine or terrestrial sediments and should circumvent any potential miscorrelations with BT or PT deposits. Found in all three ice cores with high shard concentrations and dated to 17 326 ± 319 a b2k (Table 1; Fig. 2), this tephra has huge potential as a time‐synchronous marker horizon for an interval that often poses dating challenges. For the ice, GS‐2.1 has few match points between ice cores, and this new tephra horizon adds a reliable tie‐point to synchronize cores and to facilitate GICC05 timescale transfer from NGRIP to NEEM (Fig. 5) (e.g. Rasmussen *et al*., 2013). For marine records, this common tephra deposit provides a new fix‐point in age models and also has the potential to improve assessments of variable marine reservoir offsets during the deglaciation period. This GS‐2.1 tephra is a valuable addition to the few available and well‐constrained marine‐ice tie‐points for the deglaciation period. For future use, we propose a new name for this deposit − GS‐2.1‐RHY − based on its position in the Greenland stratigraphic framework and its geochemical composition.

## Conclusions

Adopting a contiguous ice‐core sampling approach has provided further insight into the complexity of the Borrobol Tephra. Two cryptotephra deposits detected in GI‐1e ice probably equate to the BT found in terrestrial records but a firm correlation is precluded given the indistinguishable composition and closely timed deposition of the BT and PT. In this study trace element compositions show possible but tentative signs that may prove fruitful for future discrimination purposes. If these deposits are to be used as valuable marker deposits, further work is urgently required in this area. As yet there are no trace element analyses from terrestrial records that contain both the PT and the BT, and this is essential if differences are to be observed between these deposits. Re‐analysis of BT and PT major element signatures with improved microprobe operating conditions may also prove beneficial to tease out any subtle differences that may be obscured by analytical noise. Furthermore, ultra‐high‐resolution sampling of Scottish Lateglacial sequences together with high‐precision chronologies may prove beneficial to unpick the diffuse tephra profiles associated with the BT. Lastly, the GS‐2.1‐RHY horizon identified in three ice cores illustrates the value of marine‐ice tie‐points in an interval plagued by dating uncertainties and highlights its potential to assess marine reservoir offsets for the North Iceland Shelf.

## Supporting information

Supporting information relating to this article can be accessed via the publisher's website.

**Table S1.** Major oxide concentrations and secondary standards.

**Table S2.** Trace element data.
